# 1863. Pediatric Nontuberculous Mycobacteria (NTM) Lymphadenitis: an Evolving Epidemiological Pattern in Northeast Ohio

**DOI:** 10.1093/ofid/ofad500.1691

**Published:** 2023-11-27

**Authors:** Aaron Ackerman, Ankita P Desai, Jay Shah, Matthew Gropler

**Affiliations:** University of Minnesota, Minneapolis, Minnesota; University Hospitals Rainbow Babies and Children's Hospitals, Cleveland, Ohio; Department of Otolaryngology, UH Rainbow Babies and Children’s Hospital, Cleveland, Ohio; Department of Otolaryngology, UH Rainbow Babies and Children’s Hospital, Cleveland, Ohio

## Abstract

**Background:**

This study describes the changing pattern of non-tuberculous, or atypical, mycobacteria (NTM) lymphadenitis over the past five years in Northeast Ohio and analyzes factors that may be associated with this change. Particular attention will be paid to any demographic or geographic associations that can be linked with this pattern, to assess for any changing epidemiology.

**Methods:**

A retrospective chart review was completed including patients presenting to our tertiary medical center over the past five years. Patients with clinical presentation of lymphadenitis in the context of laboratory documented nontuberculous mycobacterial infection, or presumed infection, were included in the review.

**Results:**

Thirteen patients with NTM lymphadenitis presenting in Northeast Ohio were identified during the five years from 2017 to 2022. Three patients (23.1%) presented between 2017 and 2019, while ten patients (77%) presented between 2020 and 2022. The median age on presentation was 16 months old (range of 12 to 40 months). Six were male (46.2%) and seven were female (53.8%). Nine (69.2%) had positive wound cultures for Mycobacterium avium complex (MAC) and four (30.8%) were managed presumptively. Four (30.8%) were managed with medication alone, four (30.8%) were managed with surgical excision alone, and five (38.5%) were managed with both medication and surgical excision. Seven (53.8%) were treated with rifampin and azithromycin, one was treated with clarithromycin and rifabutin, and one was treated with azithromycin and ethambutol. Eight (61.5%) underwent surgical excision. Five (38%) lived within 4 miles of Lake Erie and 8 (62%) lived within 7.5 miles of Lake Erie.

**Conclusion:**

Thirteen cases of NTM lymphadenitis were identified over a five-year period in Northeast Ohio. The incidence increased from the years 2017-2019 (23.1%, n=3) to 2020-2022 (77%, n=10). Of the patients, 30.8% (n=4) responded to medication alone and 30.8% (n=4) responded to surgical excision alone, with 38.5% (n=5) requiring both medication and surgical excision. Medical therapy included combination therapy with macrolide in all cases. It is unclear if the geographic proximity to Lake Erie is contributing to the cases of NTM lymphadenitis in our area, but this needs to be further elucidated.

**Disclosures:**

**All Authors**: No reported disclosures

